# A Thermotolerant Variant of Rubisco Activase From a Wild Relative Improves Growth and Seed Yield in Rice Under Heat Stress

**DOI:** 10.3389/fpls.2018.01663

**Published:** 2018-11-20

**Authors:** Andrew P. Scafaro, Brian J. Atwell, Steven Muylaert, Brecht Van Reusel, Guillermo Alguacil Ruiz, Jeroen Van Rie, Alexander Gallé

**Affiliations:** ^1^Bayer CropScience SA-NV, Innovation Center Ghent, Ghent, Belgium; ^2^Department of Biological Sciences, Macquarie University, Sydney, NSW, Australia

**Keywords:** Rubisco activase (Rca), heat stress, *Oryza sativa* (rice), *Oryza australiensis*, yield, photosynthesis, wild relatives, thermotolerance

## Abstract

Genes encoding thermostable variants of the photosynthesis heat-labile protein Rubisco activase (Rca) from a wild relative *Oryza australiensis* were overexpressed in domesticated rice (*Oryza sativa*). Proteomics was used to quantify the abundance of *O. australiensis* Rca (Rca-*Oa*) in the resulting plants. Plants were grown to maturity in growth rooms and from early tillering until immediately prior to anthesis, they were exposed to daytime maximum temperatures of 28, 40, and 45°C and constant night temperatures of 22°C. Non-destructive measurements of leaf elongation and photosynthesis were used to compare the *null* segregant with a transfected line in which 19% of its total Rca content was the recombinant *O. australiensis* Rca (T-*Oa*-19). Height, fresh mass, panicle number, seed set, and seed number were measured at final harvest. Traits at maturity after heat stress at 45°C correlated strongly with recombinant protein abundance. Seed number was far the most responsive trait to an increase in Rca-*Oa* abundance, improving by up to 150%. Leaf elongation rates (*LER*) and tiller number were significantly greater in the transformed plants in the first two weeks of exposure to 45°C but tiller numbers later became equal in the two genotypes. Gas exchange measurements showed that T-*Oa*-19 had faster light induction of photosynthesis but not significantly higher CO_2_ assimilation rates, indicating that the carbon gain that resulted in large yield improvement after growth at 45°C was not strongly correlated with an instantaneous measurement of steady-state photosynthesis. When plants were grown at 40°C daytime maximum, there was no improvement in the final biomass, panicle or seed number when compared with 28°C, indicating that the threshold for heat damage and beneficial effects of the thermostable Rca recombinant protein was between 40 and 45°C, which corresponded to leaf temperatures in the range 38–42°C. The results suggest that the thermotolerant form of Rca from *O. australiensis* was sufficient to enhance carbohydrate accumulation and storage by rice over the life of the plant, dramatically improving yields after exposure to heat throughout the vegetative phase.

## Introduction

There is a growing expectation that improving photosynthetic potential in crops is required to boost crop productivity (Long et al., 2006; Ainsworth and Ort, 2010; Parry et al., 2011; Evans, 2013; Hermida-Carrera et al., 2016). In all plants, photosynthesis is inhibited as leaf canopies are heated beyond an optimum temperature, as determined by the climatic regime in which each species evolved (Berry and Bjorkman, 1980; Yamori et al., 2013). The rise in mean temperatures and in the duration and frequency of heatwaves in many of the world’s arable cropping regions is therefore already having a negative impact on crop yield (Lobell et al., 2011; Hartmann et al., 2013).

The introgression of genes that code for abiotic stress tolerance mechanisms into commercial crop plants offers the genuine prospect of sustained yields in adverse growing conditions (Atwell et al., 2014; Mickelbart et al., 2015; Palmgren et al., 2015). In pursuit of this aim, we have previously discovered that wild relatives of domesticated rice (*Oryza sativa*) are more heat tolerant and determined some of the physiological and biochemical factors that confer this tolerance (Scafaro et al., 2010, 2012, 2016). While many genes whose products are vulnerable to instability at high temperatures might have evolved novel isoforms in these wild species under the forces of natural selection, Scafaro et al. (2010) identified Rubisco activase (*RCA*) as a prime candidate for further investigation because heat enhanced its expression in an Australian wild species of rice (*Oryza meridionalis*). Moreover, there is evidence that the Rca protein, its post-translational regulation or aspects of its catalytic behaviour are intolerant to temperatures much above 30°C (Salvucci and Crafts-Brandner, 2004; Carmo-Silva and Salvucci, 2011; Busch and Sage, 2017).

In biochemical studies (Scafaro et al., 2016), we previously demonstrated that the form of Rca in the wild species *Oryza australiensis* has 19 amino acid changes in comparison to Rca from *O. sativa*. Furthermore, we showed that this altered protein catalysed the activation of Rubisco at temperatures up to 42°C while the efficacy of Rca from *O. sativa* became defective at temperatures exceeding 36°C. The susceptibility of rice Rca to temperatures above 35°C is supported by another recent study on Rca temperature sensitivity *in vitro* (Shivhare and Mueller-Cajar, 2017). However, these observations and others on variant forms of Rca in plants (Feller et al., 1998; Barta et al., 2010; Carmo-Silva and Salvucci, 2011) are based on *in vitro* analyses while the conditions under which Rca functions *in vivo* are poorly understood and complicated by the fact that Rca thermostability is highly dependent on concentration-dependent self-association, the presence of stabilising co-factors such as nucleotides, and ions such as Mg^2+^ (Wang et al., 1993, 2017; Henderson et al., 2013; Keown and Pearce, 2014). The role played by chaperones such as Cpn60 in the interaction between Rca and Rubisco is another unresolved factor (Salvucci, 2007), with many heat-shock proteins and associated factors expressed in heat-treated plants, and their roles as chaperones and thus stabilisers of proteins such as Rca cannot be discounted (Katiyar-Agarwal et al., 2003; Murakami et al., 2004).

Experiments were therefore designed to report on the phenotype of rice that had been transformed with various constructs designed for overexpression of the *O. australiensis RCA* gene and exposed to long-term heat through the vegetative phase of growth. A previous report in the model species *Arabidopsis thaliana* demonstrated that improvements to *in planta* Rca thermostability through genetic engineering can boost photosynthesis, growth, and development when heat stress is imposed (Kurek et al., 2007). Similarly, overexpressing a maize *RCA* in rice led to increased photosynthetic performance for rice grown at higher temperatures, particularly under non-steady-state conditions (Yamori et al., 2012). However, to our knowledge this is the first report of a transgenic crop incorporating a single *RCA* gene to test growth and developmental heat tolerance. Our hypothesis was that small *instantaneous* improvements in photosynthetic carbon gain in the transgenic plants exposed to heat will cumulatively result in major impacts on yield. Specifically, we propose that plants that fix CO_2_ more efficiently during successive weeks of vegetative growth during high temperature can subsequently re-mobilise these carbohydrates into grain. The impact of a variant form of *RCA* throughout the lifecycle is not predictable *a priori* and thus long-term observations of these aspects of plant performance in transgenic plants are essential. In this study, the efficiency with which the *RCA* transgene was expressed was estimated using multiple reaction monitoring high-resolution proteomics (MRM-HR), thus enabling correlations between the phenotypic responses and the level of *O. australiensis* recombinant Rca abundance and total Rca protein abundance.

By observing plants growing at optimal and supra-optimal temperatures through various stages of phenology, these experiments provide the most credible evidence for the role of Rca in heat tolerance and the potential for improvements through the use of variants of the *RCA* gene from wild species. Plants were grown at day temperatures of 28, 40, and 45°C but constant night temperatures of 22°C, with the higher day temperatures imposed after a four-hour ramping period. Early non-destructive observations of *LER* and tillering determined the effects of the transgene during exponential growth. Gas exchange was used to demonstrate the effect of the transgene on photosynthesis. Plants were then returned to optimal temperatures before flowering in order to prevent heat damage during meiosis. Carbon mobilisation and grain filling therefore occurred under optimal temperatures, after which numbers of panicles and tillers as well as seeds set and harvested are reported.

## Materials and Methods

### Plant Material

*Oryza sativa* ssp. *indica* cv. BHRI-43 was the parent line in all experiments. Transgenic rice (T3 homozygous Rca-*Oa* single-copy lines as described below) and segregating lines not containing the transgenes (referred to as the *null* segregant) were sown in 66-well sowing trays after seeds had been soaked in water for 1.5 days. The seedlings were transplanted to 9 cm diameter pots after 20 days and 14 individuals of similar size/height (visual score) were chosen for experiments. Plants were grown in a growth medium as outlined in Supplementary Table S1. Plants were initially grown at 28/22°C in the light/dark with a 12-h photoperiod [ca. 300 μmol m^-2^ s^-1^ photosynthetic active radiation (PAR)]. Thirty days after sowing, plants were either kept at 28°C during daytime or temperatures were ramped up over a 4-h period in the mornings to 40 or 45°C, held at this temperature for 6-h and then returned to the dark at 22°C after a 4-h ramping down. Relative humidity was kept between 60 and 70% throughout the experiments. The cycle was continued until the onset of anthesis, corresponding to a heating treatment of 99 days. A random block design was used, with the genotypes randomly assigned to positions within 10 specially separated blocks across the growth rooms to account for any confounding effects associated with growing position. Wild type plants were also grown across the borders of the growing bench to remove any influence of edge effects.

### Transformation

The coding sequences for the short Rca-β (NCBI Accession KR871003) and long Rca-α (NCBI Accession KR871002) isoform from *O. australiensis* were obtained by chemical DNA synthesis and were adapted to rice codon usage. The following three genes were inserted into a cloning vector: (1) the complete sequence of Rca-β; (2) a 5-amino acid truncated sequence of Rca-β with the last five codons deleted (i.e., AAPSS deleted from the C-terminus of the encoded protein); and (3) the complete sequence of Rca-α. In all three vectors, the gene was driven by the promoter of Rca-α from *Oryza meridionalis*. The intermediate cloning vectors were constructed in *Escherichia coli* and the final vector transferred to the acceptor *Agrobacterium tumefaciens* strain via heat-shock. All transformation events for the three vectors were generated using the same procedure. Agrobacterium-mediated gene transfer of the T-DNA vectors resulted in transfer of the DNA fragment between the T-DNA border repeats to the plant genome. As target tissue for transfection, immature embryo or embryo-derived callus were cut into small pieces, essentially using the technique described in D’halluin and Göbel (1991). Agrobacterium was co-cultivated with the rice tissues for some days, and then removed by suitable antibiotics. Transformed rice cells were selected by addition of glufosinate ammonium (with phosphinothricin 5 mg/L) to the rice tissue culture medium. Calli growing on media with glufosinate ammonium were transferred to regeneration medium. When plantlets with roots and shoots had developed, they were transferred to soil, and placed in a greenhouse. Initially 15 independent single copy transformation lines were created for each vector (i.e., 15 transformation lines for the above-described vectors 1, 2, and 3). A further gene expression analysis of seven lines for each of the three vectors was undertaken (Supplementary Figure S1) and a single line for each vector selected for the experiments as presented. A segregating line (which also underwent the transformation process) was used as the control *null* segregant. No difference in phenotype and phenology was observed among different *null* segregants when grown under standard conditions (data not shown).

### Extraction and Quantification of Recombinant Protein

Protein from leaf tissue was extracted from at least three biological replicates of each transformation line. Mature leaf blades were harvested and stored at -80°C then ground to a fine powder in a mortar and pestle in the presence of liquid nitrogen. Fifty mg of powdered leaf growing zone was immediately suspended in 1.5 mL of 10% trichloroacetic acid in acetone and 0.07% β-mercaptoethanol then incubated at -20°C for 1 h. After centrifugation for 30 min at 16000 ×*g*, the resulting pellet was washed with 1.5 mL of 100% acetone followed by centrifugation for 15 min at 16000 ×*g*. The acetone washing step was repeated three times for the complete removal of pigments, lipids and other lipophilic molecules. The colourless resulting pellet was lyophilised in a vacuum centrifuge and resuspended with 400 μL of 2% SDS in 50 mM Tris–HCl (pH 8.8). Each sample was reduced with dithiothreitol (10 mM DTT), alkylated with iodoacetamide (20 mM IAA) and then digested with 0.4 μg trypsin (1:25) for 16 h at 37°C. The digested sample was dried and resuspended in 50 μL of loading buffer (2% acetonitrile 0.1% formic acid).

### Targeting Species-Specific Peptides

An initial independent data acquisition (IDA) dataset was acquired at the Australian Proteome Analysis Facility (APAF), allowing the identification of target peptides for later scheduled high resolution multiple reaction monitoring (sMRM-HR) analysis. Various target peptides based on translated sequences were used to identify the species-specific Rca isoforms within each transgenic and *null* genotype extract. The most reliable peptides to discriminate the α and β Rca isoforms were SFQCELVFAK for *O. sativa* and SFQCELVFSK for *O. australiensis*. 10 μL (2 μg) of each digested sample was transferred to HPLC vials for MRM-HR analysis. The product ion variation window was +/-0.05 Da for peak integration. All samples were freshly prepared for each run.

For data acquisition, the sample was injected onto a peptide trap (Bruker peptide Captrap) for pre-concentration and desalted with 0.1% formic acid, 2% ACN, at 5 μL min^-1^ for 5 min. The peptide trap was then switched into line with the analytical column (Halo C_18_, 100 mm × 150 μm, 160Å, 2.7 μm). Peptides were eluted from the column using a linear solvent gradient from mobile phase A: mobile phase B (98:2) to mobile phase A: mobile phase B (65:35) where mobile phase A is 0.1% formic acid and mobile phase B is 99.9% ACN/0.1% formic acid at 600 nL min^-1^ over a 95 min period. After peptide elution, the column was cleaned with 95% buffer B for 10 min and then equilibrated with 98% buffer A for 15 min before the next sample injection. The reverse phase nanoLC eluent was subjected to positive ion nanoflow electrospray analysis. The product ion scans were 100 ms in the mass range m/z 100–1500 with the total cycle time of 1.4 s. Data were processed by MultiQuant (AB Sciex, v 2.1.1) software.

Four ^13^C- and ^15^N-labelled SIL (stable isotope labelled) peptides were used in a subsequent experiment to quantify the RCA isoforms in transgenic plants. The peptide sequences used are shown in Supplementary Table S2, with asterisks denoting the position of the labelled amino acids as follows: K^∗^ = Lys U-^13^C6; U-^15^N2 and F^∗^ = Phe U-^13^C9; U-^15^N. Each vial of SIL peptides (1 nmol/vial) was suspended in 500 μL of MQ water. The resuspended four SIL peptides were then mixed, resulting a mixed SIL peptide stock solution with a concentration of 0.5 pmol μL^-1^ for each SIL peptide. 10 μL of this mixed SIL peptide solution was added into each 10 μg rice protein sample. The concentration factor for SIL peptides was 500 pmol mg^-1^. Further mass spectrometry was performed on the TripleTOF 6600 (Sciex) and data analysis as described above.

### Vegetative and Reproductive Measurements

After the first plant started to flower, daytime temperature was reduced to 28–29°C (control conditions) in order to avoid pollen sterility. All plants were harvested at full maturity (at the completion of seed filling) and number of tillers, panicles and seeds as well as seed set (an estimated % of panicle filled with seed), above-ground fresh and dry mass (g) per plant were measured. Tiller numbers were also recorded 2 and 6 weeks after the heating as indicated in the text. Dry mass was taken after drying all above ground biomass for 1 week at 30°C until completely dry. The *LER* were measured in the second week after heating began, using a linear variable displacement transducer, essentially as described by Scafaro et al. (2016). Five individual plants from separate blocks were measured at varying times of the diurnal cycle.

### Gas Exchange Measurements

Net photosynthesis (*A*_n_) was measured using the open infra-red gas exchange system (IRGA) LI-6400 (LI-COR Inc., Lincoln, NE, United States). All measurements were made at a CO_2_ reference value of 400 μmol mol^-1^ air and a cuvette air temperature set to the prevailing growth temperature. *A*_n_ measurements were taken during the 45°C heat exposure 15–19 days after the beginning of heat exposure, 45–49 days after sowing, and all measurements were done between 12- and 16-h into the daily cycle, during the 45°C daily maximum. Plants were initially covered in plastic non-transparent boxes for 30 min to reduce PAR to less than 30 μmol m^-2^ s^-1^. The IRGA chamber was then attached to healthy fully expanded leaves with PAR turned off and automatic logging every 10 s. After the first two measurements, the PAR value was set to 1500 μmol m^-2^ s^-1^ until photosynthesis reached a steady-state. Steady-state photosynthesis was taken as the final averaged eight values measured after 40 min of exposure to a PAR of 1500 μmol m^-2^ s^-1^. The transformation of *A*_n_ and linear analysis to generate the activation state constant (*K*_a_) was the same as that outlined by Hammond et al. (1998) and Yamori et al. (2012). Seven biological replicates were measured for each genotype.

### Statistical Analysis

All statistical analysis was carried out using either GraphPad Prism 5.0 software (GraphPad Prism Software Inc., San Diego, CA, United States) or R programming language^1^ (R Core Team, 2017) Paired *t*-tests grouped by block were used for analysis of tiller number comparisons between the *null* and T-*Oa*-19 genotypes at separate time intervals over the heating treatment. Similarly, paired *t*-tests were used for fresh mass, panicle number and seed number comparisons between the *null* and T-*Oa*-19 genotypes for each of the three temperature treatments. A two-way ANOVA was used for comparisons of *LER* between the *null* and T-*Oa*-19 genotypes and across the diurnal cycle. For analysis of the interaction of transgene abundance versus relative changes in traits, a linear regression analysis was performed on all individual measurements. Boxplots show the 5–95 percentile range as a box, the minimum and maximum values as whiskers and the median as a line. Differences were considered significant at a *p*-value of less than 0.05. The mean and standard deviation for all vegetative and reproductive variables measured for all genotypes and growth temperatures are presented in Supplementary Tables S3–S5. Differences between the genotypes were analysed by a one-way ANOVA and reported as superscript letters in the Supplementary Tables.

## Results

### Correlating Phenotype With Recombinant *O. australiensis* Rca Abundance

**FIGURE 1:**
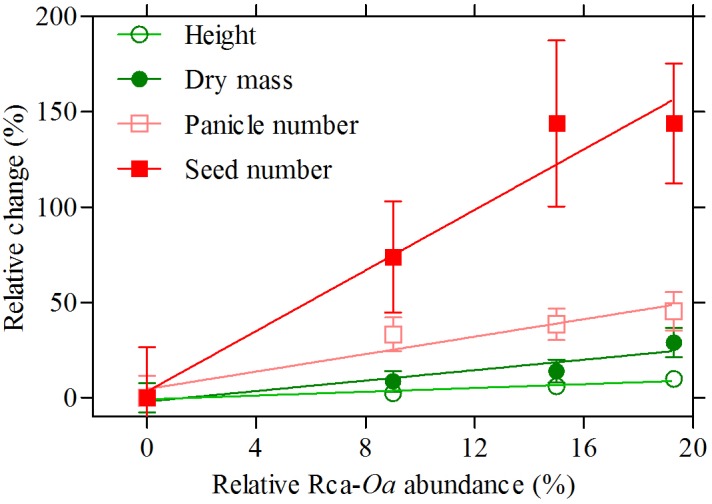
Percentage increase in four variables associated with growth and development in a *null* segregated genotype (*O. sativa*) and the Rca-*Oa*-9, Rca-*Oa*-15, and Rca-*Oa*-19 genotypes that include constructs based on the *RCA* gene from *O. australiensis*. Relative Rca-*Oa* abundance reports the degree of transgene product in plants grown under a 45°C daytime heat treatment. Linear regression analysis showed a significant positive correlation for each variable as indicated by the solid lines and reported in Table 1. Values are the mean and standard error of mean (SEM) of 14 biological replicates.

Three Rca genes from *O. australiensis* were transformed into rice, the almost identical variants Rca-β and Rca-α and a 5 amino acid truncated version of Rca-β, all known to be of similar thermostability. As such, we treated the various events from the three transformations all as lines expressing an equally thermostable Rca-*Oa* product, with the critical difference being the abundance of the recombinant protein observed for each line. The relative abundance of Rca-*Oa* as a percentage of the total Rca content *in planta* were 9.0 ± 0.01, 15.0 ± 0.02, and 19.3 ± 0.02 for the Rca-α, Rca-β, and Rca-β truncated lines, respectively, which we henceforth refer to as Rca-*Oa*-9, Rca-*Oa*-15, and Rca-*Oa*-19, with the numbers referring to the percentage content of the recombinant protein. A similar pattern in gene expression of *O. australiensis* Rca to that of the protein abundance was observed (Supplementary Figure S2) giving support to the quantitative proteomics. No significant difference (*p* = 0.42) in total Rca content (i.e., endogenous and recombinant protein combined) was detected between the *null* segregant and transgenic lines containing Rca-*Oa*: Rca made up 7.5% of total leaf protein. Plotting Rca-*Oa* abundance against four distinct traits of growth and development (Figure 1), we established that the transgene was responsible for various degrees of improvement in phenotype in plants grown under hot conditions. The two measures that best reflect vegetative growth after continuous 45°C days – plant height and dry mass (*DM*) – increased by up to 25% by maturity. Panicle number, as a measure of development, increased by about 40% because of the transgene, while the ultimate reproductive measure of plant performance in rice (seed number) was improved by more than 150% after the preceding 99 days of 45°C maxima. Positive correlations were highly significant in all cases, although *r*^2^ of the regression of the four means was modest, with values of 0.11, 0.14, 0.18, and 0.21 for plant height, *DM*, panicle number, and seed number, respectively (Table 1). These growth and reproductive variables were analysed using a correlation matrix (Table 2). A strong correlation was observed between *DM* and panicle number (*r* = 0.69). Panicle number in turn correlated strongly with seed number (*r* = 0.77). Seed number correlated positively with other measures of growth and development, namely plant height (*r* = 0.69) and seed set (*r* = 0.61), respectively. The data that follow document the strongest expressing genotype, in which Rca-*Oa* represented 19% of the Rca pool (line T-*Oa*-19) but absolute values of measured traits for all genotypes and the three growth temperatures of 28, 40, and 45°C are listed in the Supplementary Tables S3–S5.

**Table 1:** Linear regression analysis of the relationship between transgene protein abundance and growth and developmental variables as presented in Figure 1 for the null Rca-*Oa*-9, Rca-*Oa*-15, and Rca-*Oa*-19 genotypes.

	*n*	Slope	*r*^2^	*p*
Plant height	56	0.5	0.11	0.012
Dry mass	56	1.4	0.14	0.005
Panicle number	55	2.3	0.18	0.001
Seed number	53	8	0.21	0.001

*Note that the linear regression was performed on individual measurements of each genotype not the mean values as presented in Figure 1.*

**Table 2:** Pearson correlation coefficient matrix of growth and developmental variables for the null, Rca-*Oa*-9, Rca-*Oa*-15, and Rca-*Oa*-19 genotypes when grown at the 45°C heating cycle and independent of genotype (*n* = 56).

	Height	Dry mass	Panicles	Seed set	Seeds
Tillers	0.08	0.36	0.28	-0.03	0.11
Height		0.51	0.50	0.34	**0.69**
Dry mass			**0.69**	0.23	0.42
Panicles				0.49	**0.77**
Seed set					**0.61**

*Values in bold and underlined highlight r > 0.6.*

### Diurnal and Shorter-Term Implications of Heat on Growth and Development

**FIGURE 2:**
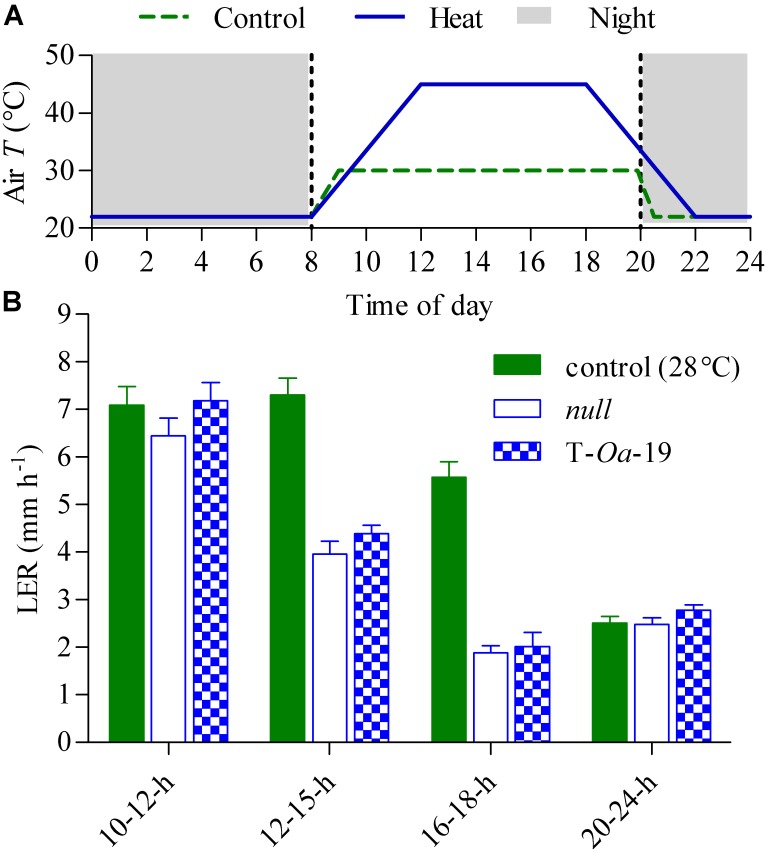
Leaf elongation rates (*LER*) of *null* and T-*Oa*-19 plants grown at constant (28°C) daytime temperatures or a six-hourly 45°C heat treatment. Data were collected from plants grown at 28°C immediately prior to imposing the heat treatment. **(A)** Representation of the 24-h diurnal cycle applied from 4 weeks after sowing until the onset of anthesis. The control (dashed green line) and 45°C treatment (solid blue line) show the changes in air temperature over the day/night cycle, with the night represented by grey shading. **(B)** *LER* are shown at four times in the diurnal cycle. Rates were identical for both genotypes at 28°C at every measurement interval (see green filled bars). *LER*s are shown alongside for the two genotypes grown at 45°C daily maximum, with open blue bars representing the *null* control and T-*Oa*-19 the most strongly expressed transgenic line (hatched bars), as seen in Figure 1. The period over which *LER* was measured appears on the *x*-axis of panel **(B)**. Values are the mean and SEM of 5–10 biological replicates. Analysis by ANOVA shows *LER* declined significantly over the diurnal cycle (*F* = 132, *p* < 0.001), and there was a significant difference in *LER* between the *null* and T-*Oa*-19 lines (*F* = 4.4, *p* = 0.0045) over the entire diurnal period.

**FIGURE 3:**
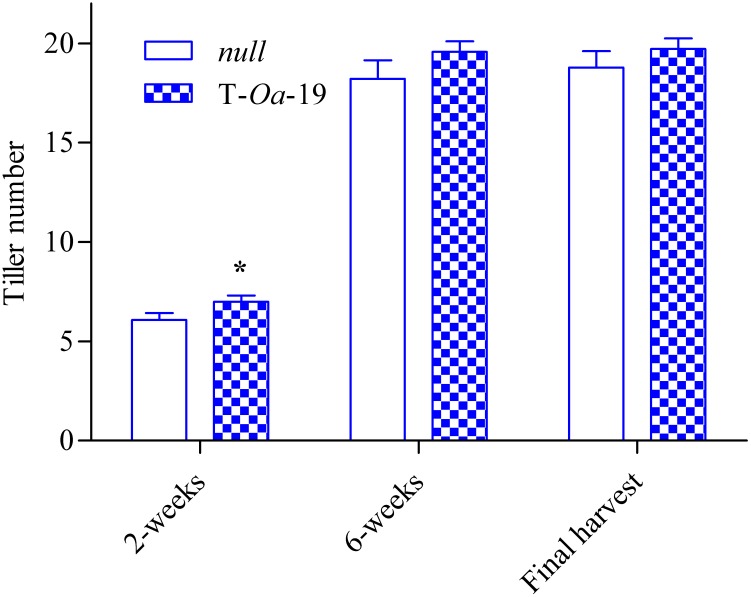
The number of tillers for the null and T-*Oa*-19 transgenic line grown under 45°C daytime maximum temperatures and counted at 2 and 6 weeks after heat imposition, and at final harvest. The asterisk indicates significance at a *p*-value of 0.05. Values are the mean and SEM of 14 biological replicates.

To capture the full impact of the diurnal temperature cycle (Figure 2A), *LER* of *null* segregant and the T-*Oa*-19 transgenic line were measured at differing times of the light period and in the dark (Figure 2B). Even when plants were maintained at maximum temperatures of 28°C, *LER* in the dark was less than a third of the rate the following morning. During the first four hours of the light period when the temperature rose by almost 6°C h^-1^, *LER* was scarcely affected by heat whereas constant exposure to 45°C dramatically reduced *LER* of both the *null* segregant and T-*Oa*-19 plants by at least 70% compared with rates in the morning. Statistical comparisons of the effect of time-of-day and genotype on *LER* revealed no significant interaction (*F* = 0.48, *p* = 0.70). Obviously, *LER* declined significantly over the diurnal cycle (*F* = 132, *p* < 0.001). Most importantly, there was a significant difference in *LER* between the *null* and T-*Oa*-19 line (*F* = 4.4, *p* = 0.0045) as a result of consistently higher rates in T-*Oa*-19 across each measuring period. Tiller numbers were also counted two and six weeks after heat treatments began and at final harvest, providing a temporal indication of the influence of heat on tiller development (Figure 3). The T-*Oa*-19 transgenic line had significantly more tillers than the *null* segregant after 2 weeks of heat (*t* = 2.5, *p* = 0.026) but tiller numbers were equal for the two genotypes four weeks later and at final harvest (*p* = 0.14 and 0.32, respectively). The greater panicle number for T-*Oa*-19 but no difference in tiller number between T-*Oa*-19 and the *null* line at harvest, implies that the productive tiller ratio (i.e., the panicle number per tiller number) was enhanced for the T-*Oa*-19 transgenic line.

### Response of Photosynthesis to Heat

**FIGURE 4:**
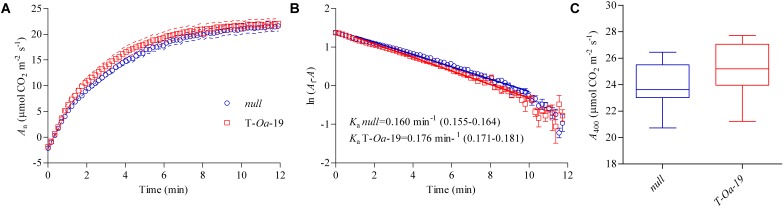
Light-induced and steady-state net photosynthesis (*A*_n_) for the null and T-*Oa*-19 transgenic line grown under 45°C daytime maximum temperatures. **(A)** After 30-min of low light (∼30 μmol m^-2^ s^-1^), high light (1500 μmol m^-2^ s^-1^) was applied and *A*_n_ recorded every 10 s. Dashed lines represent the SEM. **(B)** The log transformed mean and SEM of final *A*_n_ minus *A*_n_ at each time point (*A*_f_–*A*) was plotted against time and a linear regression fit in the range 1–10 min. The regression slope represents the Rubisco activation rate constant (*K*_a_) with the mean value given and 95% confidence interval presented in brackets. **(C)** A boxplot of the final steady-state *A*_n_ after 40 min of high light. All analyses are based on seven biological replicates.

Net photosynthetic (*A*_n_) light induction curves were measured 15–19 days into the heat application. Plants were illuminated at a PAR or 1500 μmol m^-2^ s^-1^ after previously being held in low light (∼30 μmol m^-2^ s^-1^) for 30 min. *A*_n_ induction was faster in T-*Oa*-19 plants than the *null* segregant (Figure 4A). Further analysis showed that the apparent rate constant of activation (*K*_a_) was significantly faster for T-*Oa*-19, with a rate of 0.176 min^-1^ compared with 0.160 min^-1^ for the *null* segregant (*p* < 0.001) (Figure 4B). The reciprocal of *K*_a_ shows that it took over half-a-minute longer for the *null* segregant to reach steady-state photosynthesis than T-*Oa*-19 (6.3 min *cf*. 5.7 min). These modest but significant differences as a result of the transgene are consistent with improved kinetics of Rubisco activation under hot conditions. Significantly faster rates of *non-steady-state* photosynthesis translated to higher but not statistically significant (*p* = 0.25) rates of *steady-state* photosynthesis between the two genotypes (Figure 4C).

### Temperatures Required to Obtain Benefit From the Transgene

**FIGURE 5:**
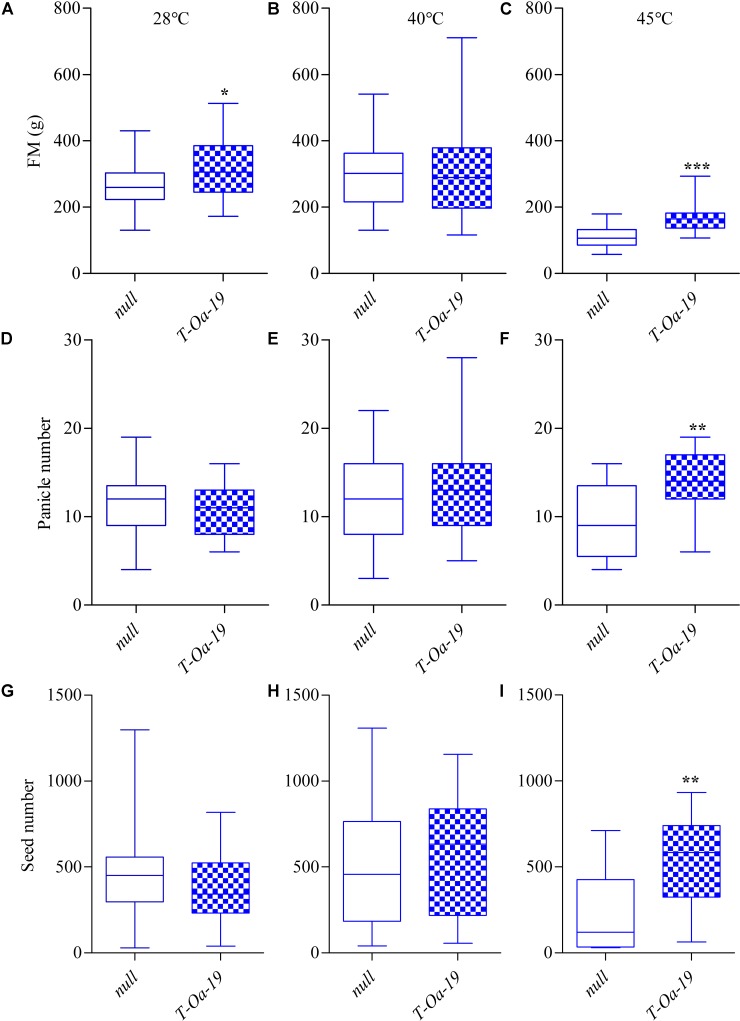
A boxplot comparison of above ground fresh mass (*FM*), panicle number and seed number for the *null* control and T-*Oa*-19 transgenic line grown at daily maxima of 28°C **(A,D,G)**, 40°C **(B,E,H)**, or 45°C **(C,F,I)**. Asterisks represent significance between the genotypes at a *p*-value less than ^∗^0.05, ^∗∗^0.01, or ^∗∗∗^0.001. Boxplots represent 16–30 biological replicates.

To determine more precisely the threshold temperature that triggers the damage we observed at 45°C air temperature, the phenotypes of the *null* and T-*Oa*-19 line were determined for plants grown to maturity after exposure to daily maxima of 28, 40, and 45°C prior to anthesis. For each of the two genotypes, aboveground fresh mass (*FM*) at final harvest was similar whether they were grown at 28 or 40°C (Figure 5), even though there was a small but significant effect of Rca-*Oa* expression on biomass in the 28°C control. However, at 45°C *FM* declined to below 200 g in both genotypes, reflecting a much-restricted rate of carbon gain. This was more pronounced for the *null* segregant, with T-*Oa*-19 accumulating significantly more biomass than the *null* segregant after 45°C maxima during the vegetative phase (*t* = 4.6, *p* < 0.001). Similarly, panicle number and seed number were not significantly different from one another at 28 and 40°C for both genotypes but again, at 45°C T-*Oa*-19 had significantly more panicles (*t* = 3.5, *p* = 0.005) and seeds (*t* = 3.3, *p* = 0.005) than the *null* segregant. Notably, seed set was also significantly greater in T-*Oa*-19 than in the *null* segregant after plants had been exposed to 45°C prior to anthesis (Supplementary Table S3). During the gas exchange measurements reported in Figure 4, leaf temperatures were shown to be 2–3°C below the daytime temperatures of 28, 40, and 45°C (data not shown). Therefore, the threshold leaf temperature at which an advantage in growth, development and reproductive success was seen as a result of the Rca-*Oa* transgene was in the range of 38–42°C.

## Discussion

It is widely agreed that as temperatures rise above 30°C, the impairment of photosynthetic activity at current atmospheric CO_2_ concentrations is in large part the result of lower Rca activity or its interaction with Rubisco (Carmo-Silva et al., 2015; Busch and Sage, 2017). Effects of altered Rca activity on non-steady state photosynthesis have been demonstrated (Hammond et al., 1998; Yamori et al., 2012; Carmo-Silva and Salvucci, 2013) but sustained faster rates of carbon assimilation seem to be more subtle and leave the question of long-term effects on plant growth in hot environments unresolved. In fact, many studies show limited direct evidence of leaf-level, steady-state photosynthesis impacting on yield (Zelitch, 1982; Fischer et al., 1998; Reynolds et al., 2000; Driever et al., 2014). In this light, a heat-tolerant form of Rca from *O. australiensis* was expressed in *O. sativa* and plant phenotype was tested over the life of the plant with a particular view to assessing yield. We demonstrate a dramatic improvement in growth and yield of heat stressed rice associated with subtle improvements in photosynthetic potential.

When expression of the Rca-*Oa* transgene was assessed by proteomics to quantify the abundance of the foreign Rca isoform in three transformed lines, it revealed a spectrum of Rca-*Oa* expression levels (9–19% of the Rca pool) and thus afforded the opportunity to calibrate phenotypic features against Rca-*Oa* expression. Of importance, the total (endogenous + recombinant) Rca pool did not change significantly among the transformed lines, so differences between lines could be attributed to the abundance of the recombinant *O. australiensis* Rca protein and not total Rca protein abundance. Previous *in vitro* analysis has demonstrated that *O. australiensis* α and β isoforms have the same thermostability profile (Scafaro et al., 2016) and the 5-amino acid truncated version did not differ from wild type Rca (unpublished results). We therefore expected no difference to exist in the thermostability characteristics of the three gene products but rather differences to occur depending on the level of overexpression. In the overexpression lines and at a 45°C daytime maximum, seed yield was the trait that was most dramatically affected, with up to a 150% (i.e., 2.5-fold) increase in numbers of seeds at maturity when 19% of the total Rca pool was present as the *O. australiensis* isoform (i.e., the T-*Oa*-19 line with the β isoform 5-amino acid truncated version). Other traits were affected less strongly, indicating that the phenotypic impact of a thermotolerant Rca after sustained heat treatment was not proportionate across all traits. For example, panicle number, which contributed directly to seed production, was also enhanced by the transgene, while traits related to biomass such as height and above ground dry mass were much less affected, and tiller numbers at maturity were not affected at all. These observations presumably reflect the manner in which additional carbon assimilation enhances the performance of this particular rice genotype; source-sink relations in cereals would predict a shifting allocation of newly acquired carbohydrates to growth, storage, respiration, and reproductive sinks (Peng et al., 1999; Yang et al., 2002; Foulkes et al., 2011). A parallel experiment in which photosynthetic carbon gain of *O. sativa* plants would be stimulated by CO_2_ enrichment at these very high temperatures would be instructive if it replicated this pattern of partitioning. Of interest, panicle numbers increased on a plot area in rice exposed to CO_2_ enrichment in FACE experiments (Yang et al., 2009) due to increased tiller number, not the productivity of individual tillers as we observed here, suggesting that temperature treatments influence carbon partitioning differently from CO_2_ enhancement.

When comparing the most strongly overexpressing line, Rca-*Oa*-19, with the *null* segregant during early phases of development soon after 45°C days were imposed, the transgene significantly enhanced leaf elongation and increased tiller number, indicating partitioning of additional photoassimilates to growth (Figures 2, 3). These stimulatory effects were in the order of 10% but were apparent in the first two weeks of heat treatment and therefore subject to amplification in the weeks of exponential growth that would follow. While *LER* is a non-destructive surrogate for growth (*cf*. biomass), these instantaneous measurements show both the slump in growth as the duration of heat continues through the day and the consistent and significant gains that are seen in leaves of the most strongly expressing Rca-*Oa*-19 line. The benefit to leaf growth was maintained at each sampling time but the impact of Rca-*Oa* on root growth and development remains to be investigated.

Similar to leaf elongation, light induction curves showed a small but significantly steeper rise in photosynthesis in Rca-*Oa* plants after illumination (Figure 4) while steady-state rates of assimilation were also higher but not significantly. This small difference in induction and limited changes in steady-state photosynthesis are expected as severe knockdown of 80% or more of Rca content is needed to elicit a dramatic difference in light induction kinetics while still having limited impact on steady-state photosynthesis (Jiang et al., 1994; Hammond et al., 1998). Furthermore, Yamori et al. (2012), who transformed rice with a maize *Rca*, also showed that light induction was responsive to the transgene while steady-state photosynthesis responded only weakly. These results would suggest that the benefit of thermostable Rca manifests itself through enhanced photosynthetic performance when photosynthesis is in flux. Considering these experiments were undertaken in controlled growth rooms, one might expect the more dynamic environment of the field to elicit an even stronger response of the Rca-*Oa* recombinant protein.

The choice of 45°C as an appropriate temperature to heat stress rice with abundant water supply was evident in that the data reveal no impact of heat on growth and development traits, including yield until daily maxima were raised to 45°C. It should be noted that leaf temperatures were 2–3°C lower than air temperature, suggesting that the threshold leaf temperature for heat-induced damage to photosynthetic biochemistry was between 38 and 42°C. This temperature threshold accords with our previous *in vitro* findings, where temperatures greater than 36°C severely impaired function of Rca-*Os* while having little effect on Rca-*Oa* (Scafaro et al., 2016). In this temperature range, the advantageous effect of Rca-*Oa* expression at levels below 20% of the total Rca complement was sufficient to enhance vegetative growth, final biomass, panicle development and, most particularly, seed number. Because the grain developed optimally in all plants at 30°C, variation in yield is reflected in seed number (Yoshida, 1981; Yoshida et al., 2006; Makino, 2011; Sánchez et al., 2014). As well as higher seed numbers, there is also evidence that seed set was positively affected by Rca-*Oa* expression, supporting the case that the transgene enhanced potential yield as reflected by seed set (Makino, 2011).

The effect of the wild rice Rca recombinant protein on the phenotype of heat-treated transgenic rice plants is complex. Part of the complexity arose through the requirement that plants had to be exposed to sufficient heat (45°C) in order to elicit a metabolic penalty during vegetative development but the heat treatment had to be discontinued once the maturity phase of reproductive development commenced in order not to affect photoassimilate mobilisation to the grain. Such a regime tested the cumulative impact of heat on accrual of photoassimilates without confounding the effect by sterilising the gametes, which is a well-known effect of heat on rice (Prasad et al., 2006). Despite removing the heat treatment at the onset of anthesis, we cannot exclude the possibility that heat affected reproductive processes prior to this point, as drought stress has been shown to increase pre-anthesis spikelet abortion in rice (Kato et al., 2008). It is argued that increased photoassimilate resource accumulation positively influences the pre-anthesis meiosis success with exposure to stresses including heat (Barnabás et al., 2008), so arguably, overexpression of the thermostable Rca-*Oa* could have conferred a benefit to reproductive development in the transgenic lines upon heat treatment during this particular period. While we prevented to a large extent the impact of gamete sterility on potential yield by discontinuing the heat stress at anthesis, the plants in this study allocated marginally more carbon to growth in the early stages of development and this cumulative effect was reflected in final biomass, which was strongly correlated with panicle number. However, tiller numbers in later development, and steady-state photosynthetic rates, did not respond to the expression of the transgene, indicating that the carbon gain in Rca-*Oa* overexpressing plants after a long heat treatment did not translate into either exaggerated development (excessive tillering) and was not perceptible through faster photosynthesis at one point in time; carbon mass-balance budgets at various stages of development would be required to discover how the additional carbon was accrued (Gregory and Atwell, 1991), as it is difficult to link instantaneous gas-exchange measurements of a single tissue type and at any point in time to final biomass and yield (Parry et al., 2011). Zelitch (1982) explored this “paradox” thoroughly and concluded from many prior studies that an alignment of rates of instantaneous CO_2_ assimilation and long-term biomass accumulation is exceedingly rare in studies of annual crop species. It seems in this case that marginally improved rates of steady-state net photosynthesis as well as induction of photosynthesis to perturbations such as light was a contributor to the additional carbon that was gained as a result of Rca-*Oa* expression. We postulate this carbon was stored in stems in the late vegetative stage, as would be predicted from classical studies of cereal development (Bell and Incoll, 1990; Blum et al., 1994; Morita and Nakano, 2011). The mobilisation of these stored carbohydrates (principally starch in rice) prior to and after flowering would then account for the very substantial gain in yield that the *O. australiensis* Rca confers on rice plants grown through most of their vegetative development in 45°C days.

## Author Contributions

AG, JVR, BA, and AS conceptualised the experiments. SM, BVR, GR, BA, and AS conducted the experiments. AS and BA wrote the initial draft of the manuscript with subsequent input from AG and JVR. AS and BA contributed equally.

## Acknowledgements

We thank Stefanie Van Laere for help with plant growth, Izabela Matyszczak for help with gas exchange measurements, Aaron Phillips for help with statistical analysis, and Nancy Van De Steene for vector construction. We also thank Rosita Le Page, Hilde Maes, and Eveline Bossier for plant transformation, and Bart den Boer for manuscript suggestions. Proteomic services were undertaken by Xiaomin Song, Thiri Zaw, and Mark Molloy at APAF with the infrastructure provided by the Australian Government through the National Collaborative Research Infrastructure Strategy (NCRIS).

## Conflict of Interest Statement

AS and BA are inventors named on a patent application pertaining to this work.The remaining authors declare that the research was conducted in the absence of any commercial or financial relationships that could be construed as a potential conflict of interest.

## Footnotes

1 https://www.R-project.org/

## Supplementary Material

FIGURE S1 Gene expression analysis of independent transgenic lines for the three genotypes T-*Oa*-9, T-*Oa*-15, and T-*Oa*-19 at F1 generation. Expression is given as the fold-change in target gene relative to a protein kinase reference gene (TIGR Identifier:LOC_Os06g48970.1).

FIGURE S2 The gene expression and protein abundance of the three *O. australiensis* Rca transgenic lines T-*Oa*-9, T-*Oa*-15, and T-*Oa*-19. Gene expression is presented as the fold-change in the gene of interest relative to a protein kinase reference gene. Protein abundance is presented as recombinant *O. australiensis* Rca as a percentage of the total Rca content (endogenous + recombinant).

TABLE S1 Nutrient solution contents used for growing rice and at final pH 5.4.

TABLE S2 SIL peptides for identification of the proportions of Rca from *O*. *sativa* and *O*. *australiensis* in protein extracts from transgenic plants. These peptides were used in combination with protein extracts in order to identify precise retention times of native peptides in mass spectra and thereby, quantify the specific Rca isoforms from each species. Charge and the Q1 ions used to identify individual peptides are shown in the table.

TABLE S3 Growth and developmental characteristics among wild type and Rca transgenic rice grown at 45°C.

TABLE S4 Growth and developmental characteristics among wild type and Rca transgenic rice grown at 40°C.

TABLE S5 Growth and developmental characteristics among wild type and Rca transgenic rice grown at 28°C.
