# Supplementary figures and images for: A Thermotolerant Variant of Rubisco Activase From a Wild Relative Improves Growth and Seed Yield in Rice Under Heat Stress
Source: Front Plant Sci. 2018 Nov 20;9:1663. doi: 10.3389/fpls.2018.01663 (PMC6256286; doi:10.3389/fpls.2018.01663)

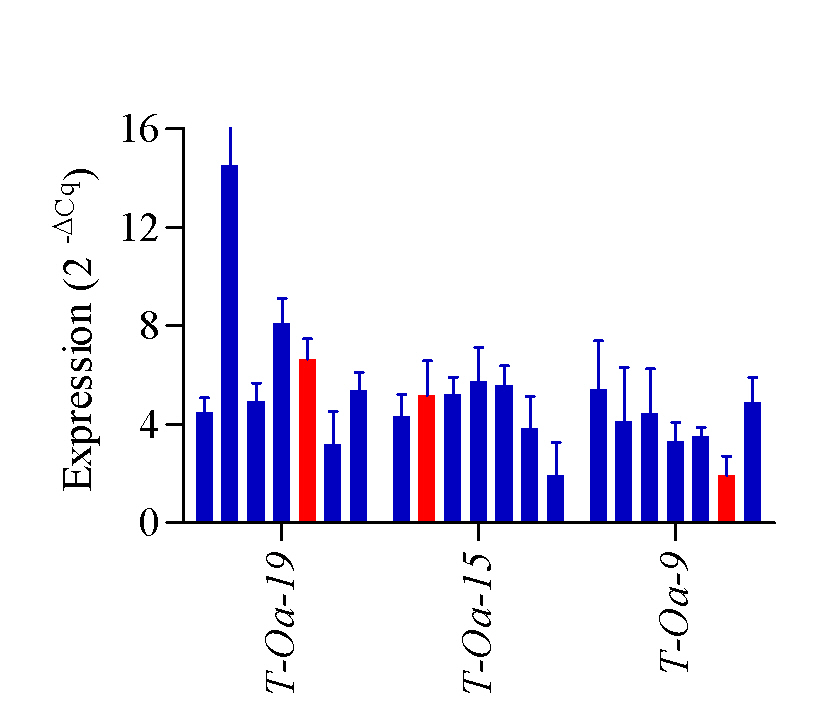

Supplement: FIGURE S1 — Gene expression analysis of independent transgenic lines for the three genotypes T-Oa-9, T-Oa-15, and T-Oa-19 at F1 generation. Expression is given as the fold-change in target gene relative to a protein kinase reference gene (TIGR Identifier:LOC_Os06g48970.1). [file Image_1.JPEG]

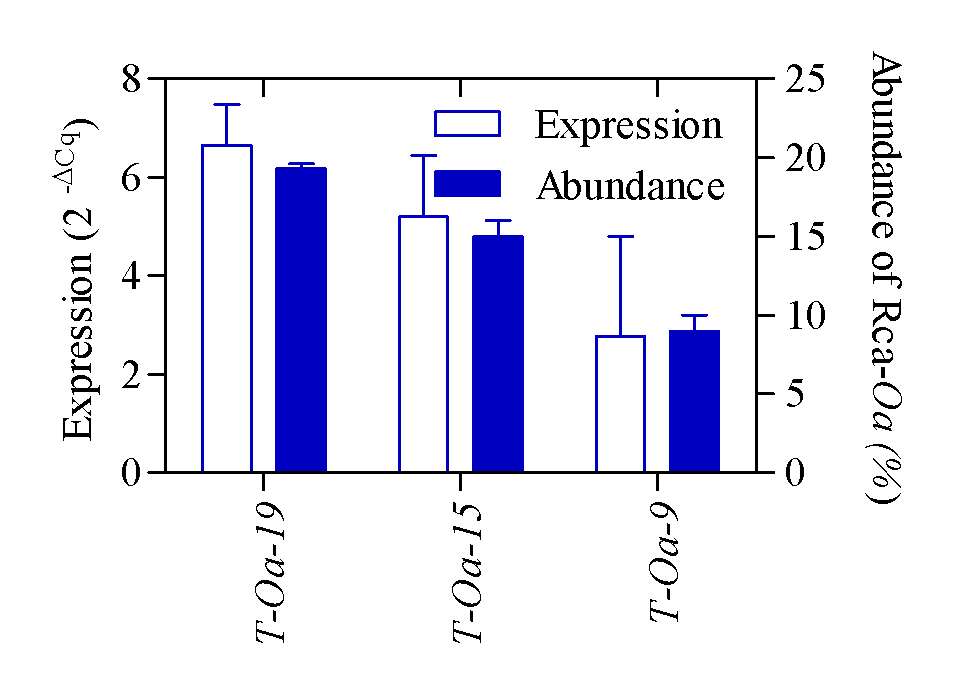

Supplement: FIGURE S2 — The gene expression and protein abundance of the three O. australiensis Rca transgenic lines T-Oa-9, T-Oa-15, and T-Oa-19. Gene expression is presented as the fold-change in the gene of interest relative to a protein kinase reference gene. Protein abundance is presented as recombinant O. australiensis Rca as a percentage of the total Rca content (endogenous + recombinant). [file Image_2.JPEG]
