# Supplementary material for: A Thermotolerant Variant of Rubisco Activase From a Wild Relative Improves Growth and Seed Yield in Rice Under Heat Stress
Source: Front Plant Sci. 2018 Nov 20;9:1663. doi: 10.3389/fpls.2018.01663 (PMC6256286; doi:10.3389/fpls.2018.01663)
Supplement: TABLE S1 — Nutrient solution contents used for growing rice and at final pH 5.4. [file Table_1.DOCX]

| **Supplementary table S1.** Nutrient solution contents used for growing rice and at final pH 5.4. | | |
| --- | --- | --- |
|  |  |  |
|  | mmol/l | mg/l |
| N-NO_3_ | 4.74 | 66.3 |
| N-NH_4_ | 0.28 | 4.0 |
| N-tot | 5.02 | 70.3 |
| CaO | 1.69 | 94.9 |
| Ca | 1.80 | 72.2 |
| P_2_O_5_ | 0.35 | 50.0 |
| P | 0.70 | 21.8 |
| K_2_O | 0.88 | 82.7 |
| K | 1.75 | 68.6 |
| MgO | 1.36 | 54.7 |
| Mg | 1.41 | 34.2 |
| SO_3_ | 1.37 | 109.5 |
| S | 1.36 | 43.8 |
|  | µmol/l | mg/l |
| Fe | 50.83 | 2.8 |
