# Supplementary material for: A Thermotolerant Variant of Rubisco Activase From a Wild Relative Improves Growth and Seed Yield in Rice Under Heat Stress
Source: Front Plant Sci. 2018 Nov 20;9:1663. doi: 10.3389/fpls.2018.01663 (PMC6256286; doi:10.3389/fpls.2018.01663)

**Supplementary table S2**: SIL peptides for identification of the proportions of Rca from *O*. *sativa* and *O*. *australiensis* in protein extracts from transgenic plants. These peptides were used in combination with protein extracts in order to identify precise retention times of native peptides in mass spectra and thereby, quantify the specific Rca isoforms from each species. Charge and the Q1 ions used to identify individual peptides are shown in the table.


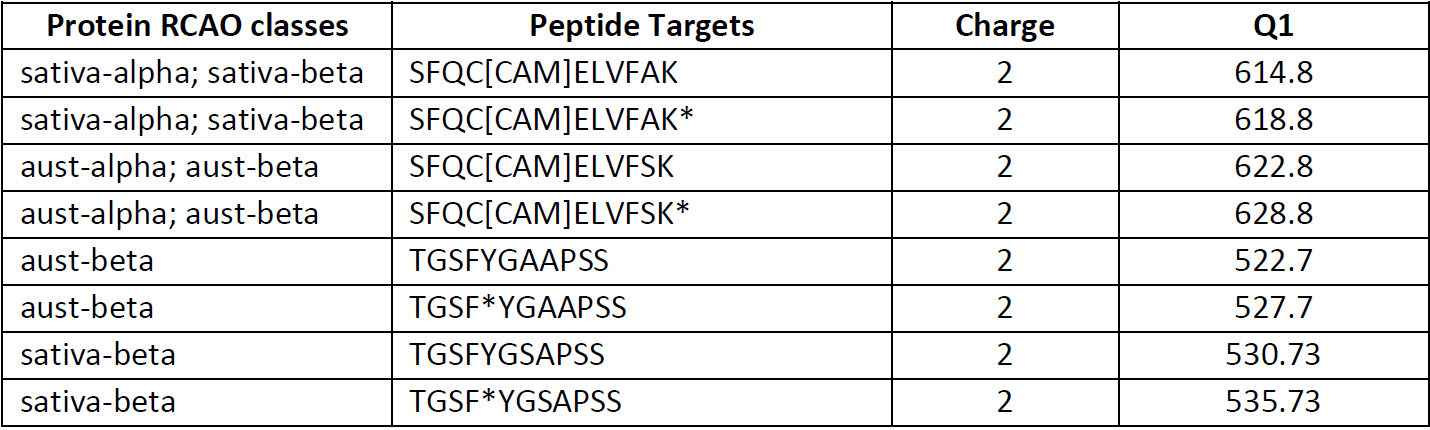

Supplement: TABLE S2 — SIL peptides for identification of the proportions of Rca from O. sativa and O. australiensis in protein extracts from transgenic plants. These peptides were used in combination with protein extracts in order to identify precise retention times of native peptides in mass spectra and thereby, quantify the specific Rca isoforms from each species. Charge and the Q1 ions used to identify individual peptides are shown in the table. [file Table_2.DOCX]
