# Supplementary material for: A Thermotolerant Variant of Rubisco Activase From a Wild Relative Improves Growth and Seed Yield in Rice Under Heat Stress
Source: Front Plant Sci. 2018 Nov 20;9:1663. doi: 10.3389/fpls.2018.01663 (PMC6256286; doi:10.3389/fpls.2018.01663)
Supplement: TABLE S3 — Growth and developmental characteristics among wild type and Rca transgenic rice grown at 45°C. [file Table_3.DOCX]

| **Supplementary table S3.** Growth and developmental characteristics among wild type and Rca transgenic rice grown at 45°C | | | | |
| --- | --- | --- | --- | --- |
|  | Line | | | |
| parameter | WT  *n*=14 | *T*-*Oa*- 9  *n*=14 | *T*-*Oa*- 15  *n*=14 | *T*-*Oa* -19  *n*=14 |
| Transgene abundance ( Rca-*Oa* as % Rca-*Os*) | 0±0^a^ | 9.0±0.01^b^ | 15.0±0.02^c^ | 19.3±0.02^d^ |
| Tillers (total number) | 19±3^a,b^ | 20±2^b^ | 16±3^c^ | 20±2^a,b^ |
| Plant height (cm) | 80±8^a,b^ | 82±7^a^ | 85±9^a,b^ | 88±9^a,b^ |
| Fresh mass (g) | 111±34^a^ | 127±35^a,b^ | 126±27^a,b^ | 168±46^b^ |
| Dry mass (g) | 42±12^a^ | 46±9^a,b^ | 48±10^a,b^ | 54±12^b^ |
| Panicle number | 10±4^a^ | 13±3^a,b^ | 13±3^b^ | 14±4^b^ |
| Anthesis (days after sowing) | 148±9^a^ | 150±6^a^ | 148±7^a^ | 148±8^a^ |
| Seed set (% filled) | 41±19 ^a^ | 55±23 ^a,b^ | 70±12 ^b^ | 61±13^b^ |
| Seed number | 220±217^a^ | 382±240^a^ | 706±441^b^ | 536±259^b,c^ |
