# Supplementary material for: A Thermotolerant Variant of Rubisco Activase From a Wild Relative Improves Growth and Seed Yield in Rice Under Heat Stress
Source: Front Plant Sci. 2018 Nov 20;9:1663. doi: 10.3389/fpls.2018.01663 (PMC6256286; doi:10.3389/fpls.2018.01663)
Supplement: TABLE S4 — Growth and developmental characteristics among wild type and Rca transgenic rice grown at 40°C. [file Table_4.DOCX]

|  |
| --- |

| **Supplementary table S4.** Growth and developmental characteristics among wild type and Rca transgenic rice grown at 40°C | | | | |
| --- | --- | --- | --- | --- |
|  | Line | | | |
| parameter | WT  *n*=30 | *T*-*Oa*- 9  *n*=40 | *T*-*Oa*- 15  *n*=35 | *T*-*Oa* -19  *n*=29 |
| Tillers (total number) | 21±6^a^ | 19±5^a^ | 17±7^a^ | 20±9^a^ |
| Plant height (cm) | 86±7^a^ | 83±10^a^ | 81±8^a^ | 84±7^a^ |
| Fresh mass (g) | 304±108^a^ | 273±97^a,b^ | 228±95^b^ | 315±153^a^ |
| Dry mass (g) | 72±31^a^ | 67±28^a,b^ | 52±23^b^ | 68±40 ^a,b^ |
| Panicle number | 13±5^a,b^ | 11±4^a,b^ | 10±5^a^ | 14±6^b^ |
| Seed set (% filled) | 33±21 ^a^ | 30±19^a^ | 33±23^a^ | 42±18 ^a^ |
| Seed number | 492±347^a^ | 390±310^a,b^ | 229±170^b^ | 566±316^a,c^ |
