# Supplementary material for: A Thermotolerant Variant of Rubisco Activase From a Wild Relative Improves Growth and Seed Yield in Rice Under Heat Stress
Source: Front Plant Sci. 2018 Nov 20;9:1663. doi: 10.3389/fpls.2018.01663 (PMC6256286; doi:10.3389/fpls.2018.01663)
Supplement: TABLE S5 — Growth and developmental characteristics among wild type and Rca transgenic rice grown at 28°C. [file Table_5.DOCX]

| **Supplementary table S5.** Growth and developmental characteristics among wild type and Rca transgenic rice grown at 28°C | | | | |
| --- | --- | --- | --- | --- |
|  | Line | | | |
| parameter | WT  *n*=30 | *T*-*Oa*- 9  *n*=35 | *T*-*Oa*- 15  *n*=40 | *T*-*Oa* -19  *n*=29 |
| Tillers (total number) | 16±4^a^ | 16±4^a^ | 13±4^b^ | 16±4^a,b^ |
| Plant height (cm) | 94±6^a,b^ | 94±6^a,b^ | 92±6^b^ | 97±7^a^ |
| Fresh mass (g) | 264±66^a^ | 268±79^a^ | 212±77^b^ | 316±89^a^ |
| Dry mass (g) | NA | NA | NA | NA |
| Panicle number | 12±4^a^ | 11±3^a,b^ | 9±3^b^ | 11±3^a,b^ |
| Seed set (% filled) | 46±16 ^a^ | 73±14^b^ | 38±20 ^a,b^ | 40±15 ^a,b^ |
| Seed number | 455±254^a^ | 288±171^b^ | 290±200^b^ | 377±202^a,b^ |
